# The effectiveness and pharmacoeconomic study of using different corticosteroids in the treatment of hypersensitivity pneumonitis

**DOI:** 10.1186/s12890-024-02896-z

**Published:** 2024-02-15

**Authors:** Marwa G. Elhennawy, Yosri Akl, Maggie Abbassi, Nirmeen A. Sabry

**Affiliations:** 1 Department of Clinical Pharmacy, Faculty of Pharmacy, Cairo University, 11562 Cairo, Egypt; 2 Department of Pulmonology, Faculty of Medicine, Cairo University, 11562 Cairo, Egypt

**Keywords:** HP, Corticosteroids, Efficacy, Cost-effectiveness analysis, Spirometry, KL-6

## Abstract

**Purpose:**

Interstitial lung diseases (ILDs) are caused by inflammation and/or fibrosis of alveolar walls resulting in impaired gas exchange. Hypersensitivity pneumonitis (HP) is the third most common type of ILDs. Corticosteroids are the mainstay treatment for HP. The use of intramuscular (IM) betamethasone or intravenous (IV) dexamethasone as weekly pulse doses has shown higher benefit than daily oral prednisolone for HP patients. The aim of this study is to directly compare different corticosteroids in terms of effectiveness and in monetary values and perform an economic evaluation.

**Methods:**

One hundred and seven patients were tested for pulmonary function tests (PFTs) and inflammatory markers to assess the treatment effectiveness. A cost-effectiveness analysis (CEA) was performed. ICERs between 3 treatment groups were calculated.

**Results:**

Post treatment, Krebs von den Lungen-6 (KL-6) levels significantly improved in betamethasone group from 723.22 ± 218.18 U/ml to 554.48 ± 129.69 U/ml (*p* = 0.001). A significant improvement in erythrocyte sedimentation rate (ESR) occurred in the dexamethasone group from 56.12 ± 27.97 mm to 30.06 ± 16.04 mm (*p* = 0.048). A significant improvement in forced expiratory volume (FEV_1_), forced vital capacity (FVC) and six-minute walk distance (6MWD) was observed within the three treatment groups. A significant improvement in oxygen desaturation percentage (SpO_2_) occurred within dexamethasone and betamethasone groups. Betamethasone and dexamethasone were found more cost-effective than prednisolone as their ICERs fell in quadrant C. Furthermore, ICER between betamethasone and dexamethasone was performed; a small difference in cost was found compared to the higher benefit of betamethasone.

**Conclusion:**

Betamethasone and dexamethasone were found to be more effective than prednisolone in improving the inflammatory reaction and the clinical features of HP patients. Betamethasone was found to be the best intervention in terms of cost against the effect.

**Supplementary Information:**

The online version contains supplementary material available at 10.1186/s12890-024-02896-z.

## Introduction

Interstitial lung diseases (ILDs) are also known as diffuse parenchymal lung diseases [1] including more than 200 heterogenous diseases [2], and their hallmark is the primary involvement of lung interstitium [3]. The main common features shared by ILDs are interstitium targeting, the possibility of lung scarring and the radiological/histopathological pattern [4].

ILDs should be diagnosed by symptoms of breathlessness due to impaired gas exchange which results from the spreading inflammation and/or fibrosis of alveolar walls [5]. Symptoms are usually supported by chest radiography and high-resolution computed tomography imaging (HRCT). Pulmonary function tests (PFTs) show restriction in ventilation and hypoxemia [5].

Hypersensitivity pneumonitis (HP) is the most common type of ILDs in Egypt [6] and the prevalence increases in at-risk populations such as farmers and bird breeders [7]. Most of the study subjects were from the rural areas where farming, animals and bird breeding is most common; hence HP was the focus of this study. HP is caused mainly by environmental insult, providing a marked benefit to the patient when stopped [8]. HP diagnosis is supported by CT scan showing diffuse ground glass or centrilobular ground glass nodules, poorly defined centrilobular nodules, areas of air trapping and fibrotic alterations as septal thickening, honeycombing and traction bronchiectasis represented in a peribronchovascular distribution predominantly in the mid and upper lung zone [9].

The objective of HP treatment is to decrease inflammation and hence the elimination of the resulting fibrosis [10]. The responsiveness to treatment, inflammation and disease progressiveness were usually assessed using HRCT and PFTs [11]. However, HRCT was found of limited value in prognosis of disease decline and survival [12] and the presence of other pulmonary conditions as emphysema or pulmonary hypertension that may be associated with HP may invalidate PFTs results [13]. Therefore, more accurate prognostic information are highly needed, and it can be provided by lung specific biomarkers [10]. One of the lung-specific epithelium proteins is Krebs von den Lungen-6 (KL-6) [14]. It is found to be a valuable biomarker associated with the prognosis of ILDs by showing the level of damage as well as the reformation of type II pneumocytes [15]. Recently, the increased levels of KL-6 serve as severity indicator and early progression detector in patients with ILDs [16].

Corticosteroids are the mainstay treatment for ILDs and so HP by the direct reduction of T-lymphocyte numbers and neutrophil adherence, moreover, the modification of macrophage function; including the decrease of tumor necrosis factor (TNF) release [17]. The primary corticosteroid for treatment of HP is prednisolone [18], however by clinical practice the use of intramuscular (IM) betamethasone or intravenous (IV) dexamethasone as weekly pulse doses has shown higher benefit for HP patients through the improvement of pulmonary functions and quality of life [19]. Although the oral route of administration is more accepted by the patients due to ease and comfort of administration; yet the pulse parenteral way of administration provides the benefit of decreased adverse drug reactions [20]. The choice of betamethasone and dexamethasone was favored over prednisolone due to the absence of their mineralocorticoid activity and weak immunosuppressive effect [21] and longer duration of action than prednisolone [22]. Although betamethasone and dexamethasone are highly similar in structure; yet betamethasone has a slightly increased anti-inflammatory effect over dexamethasone [23].

Therefore, it is worth to directly compare the three corticosteroids not only in terms of effectiveness but also in monetary values and perform an economic evaluation. Economic evaluation answers the question whether a new treatment is worth paying for if compared to other feasible uses of the same limited resources, ensuring that effectiveness has been reserved [24]. So, the questions to be raised; are dexamethasone and betamethasone more effective than prednisolone? Which drug of the three is the most cost effective? These questions remain unanswered.

## Subjects and methods

### Study design

This was a three months prospective, open label, and parallel study to test the effectiveness of three different corticosteroids in the management of HP, followed by a pharmacoeconomic analysis to find which regimen (betamethasone, dexamethasone or prednisolone) is the most cost-effective.

### Study subjects

Patients were recruited from the outpatient clinics of the Pulmonary Medicine Department, Kasr Al-Ainy Teaching Hospital, Cairo University, Egypt. Informed consent was obtained from all subjects or their caregivers after explaining the nature, purpose, and potential risks of the study. The study protocol was approved by the Ethics Committee, Faculty of Pharmacy, Cairo University (CL (2183)). The study protocol is registered on ClinicalTrials.gov (identifier: NCT04982809, 29/7/2021). Patients with HP (chronic hypersensitivity pneumonitis confirmed by HRCT) recruited were, adults patients (aged between 18 and 65 years old), having symptoms of dry cough and shortness of breath, with significant decrease in pulmonary function (desaturation by 4% after 6-minute walk distance) [25], patients who were no longer controlled (decline in forced vital capacity by more than or equal 10% [26]) on their prednisolone maintenance therapy (up to 10 mg/day) [27]. Exclusion criteria included patients with any other organ affliction (heart failure, renal failure, or previously diagnosed with liver cirrhosis, patients with active infection, or those on other immunosuppressive medications (e.g., cyclophosphamide), anti-fibrotic drugs (e.g., pirfenidone, nintednib) and other medications (e.g., interferon, endothelin-1 antagonist and tumor necrosis factor α modulator), patients with history of pulmonary embolism and pregnant patients. It has been assumed that whenever ILD patients become fibrotic, an antifibrotic treatment is needed to slow down the progression irrelevant to the initial trigger [28, 29]. However, there was no agreement when to start the antifibrotic therapy in fibrotic HP [30]. The INBUILD trial with fibrosing ILD patients other than IPF set the criteria for using antifibrotics (e.g. nintedanib) such as progression over the past two years despite the appropriate management [31]. Patients meeting these progression criteria who required antifibrotic therapy were excluded from our study.

### Clinical assessment and medications

Three different corticosteroid regimens were evaluated for the management of HP where the patients were divided into three treatment groups for three consecutive months:

- Group I: patients receiving weekly pulse doses of betamethasone IM injection.
- Group II: patients receiving weekly pulse doses of dexamethasone IV injection.
- Group III: patients receiving oral prednisolone daily (dose range 15–20 mg/day) [32].

(The pulse doses of betamethasone and dexamethasone are equivalent to that of prednisolone daily dose in a week).

10 mg oral prednisolone oral ≡ 1.5 mg dexamethasone intravenous ≡ 1.2 mg betamethasone intramuscular [33].

At baseline, all subjects were screened for their demographic data, smoking habits, medical and medication history. The monitoring parameters included PFTs (forced expiratory volume in the 1st second (FEV_1_), forced vital capacity (FVC) and the ratio between them), 6-minute walk distance test (6MWD), and percentage of desaturation using pulse oximetry. Laboratory tests were also done to measure the treatment efficacy including KL-6, erythrocyte sedimentation rate (ESR) and C-reactive protein (CRP). Safety was evaluated by measuring liver enzymes and random blood glucose together with blood pressure. All above mentioned parameters were performed at baseline and after completion of treatment. KL-6 was the primary outcome in this study while the secondary outcomes were PFTs (FEV_1_, FVC, the ratio between them), 6MWD, percentage of oxygen desaturation, ESR and CRP. Complete blood picture was performed prior to enrolment to rule out active infections.

### Cost effectiveness analysis

The study was designed from patient perspective where the direct medical costs as well as the indirect costs (e.g., loss of patients’ income) were evaluated. The costs were collected directly from the patient prospectively throughout the study period. All costs were actual coverage of the treatment expenses including the cost of the medications, the cost of the hospital stay due to HP during the study period, the cost of the physician’s visit and the cost paid by the patient in order to administer the recommended medication, any extra costs due to exacerbations or adverse effects due to the use of corticosteroids and costs of transportation and days-off.

Effectiveness was measured by comparing patients’ KL-6 levels before and after 3 months of corticosteroids treatment. The most effective medication is considered to be the one with the lowest post-treatment KL-6 levels.

The incremental cost-effectiveness ratios (ICERs) were calculated for the three corticosteroids regimens by dividing the average difference in costs by the average difference of effectiveness in terms of KL-6 levels using the following equation. Then, placed on the cost-effectiveness plane [34] to determine the most cost-effective intervention.

$$\displaylines{ICER{\text{ }}(Incremental{\text{ }}Cost{\text{ }}Effectiveness{\text{ }}Ratio) = \cr \frac{{Cost{\text{ }}A - Cost{\text{ }}B}}{{Effect{\text{ }}A - Effect{\text{ }}B}} \cr} $$

### Statistical analysis

Sample size estimation was calculated using G*Power 3.1.9.2 (University of Düsseldorf, Düsseldorf, Germany). Sample size was calculated based on the means and standard deviations of the study conducted by *Yang Hu et al.* [35] using ANOVA, Fixed effects, Omnibus, one-way statistical test assuming effect size of (F = 0.316). A total sample size of 102 patients was needed to achieve power of 80% assuming alpha = 0.05.

Statistical analyses were performed using the software package Statistical Program for Social Sciences SSPS version 25 (IBM, USA); *p* values < 0.05 were considered significant. All continuous variables were tested for normality using the Shapiro-Wilk test.

All continuous data were expressed as mean and standard deviation, while categorical data were expressed as frequency and/or percentages in Table [36]. For continuous data, one way ANOVA test was used for intergroup comparison while independent samples Kruskal Wallis test was used in categorical data. To compare the effect within the same group paired t-test was used in continuous data, Wilcoxon Signed-Rank test was used in ordinal data and McNemar test was used for nominal data. The statistical analyses were conducted by the primary investigator of the study.

## Results

### Demographic characteristics of recruited patients

During the period between July 2018 and March 2020, 107 patients fulfilled the inclusion and exclusion criteria. Most of the recruited subjects (88.79%) were females. The study subjects were distributed among the three groups of treatment where 35 patients received betamethasone injection, 33 patients received dexamethasone injection and 39 patients received prednisolone tablets.

On comparing the demographic characters of the three groups there was no statistically significant difference found as shown in Table 1.

**Table 1:** Demographic characteristics of study subjects

Parameter	Betamethasone (*N* = 35)		Dexamethasone (*N* = 33)	Prednisolone (*N* = 39)		***P***-Value*	
**Age (**years**) (**mean ± SD**)**	45.94 ± 12.11		45.21 ± 9.93	46.62 ± 11.84		0.873^Θ^	
**Weight (**Kg**) (**mean ± SD**)**	78.39 ± 19.66		79.21 ± 15.58	78.95 ± 20.22		0.987^Θ^	
**Height (**cm**) (**mean ± SD**)**	158.46 ± 8.97		160.17 ± 6.31	158.36 ± 8.04		0.647^Θ^	
**Disease Duration (**years**) (**mean ± SD**)**	3.87 ± 2.53		4 ± 3.39	4.24 ± 5.04		0.928^Θ^	
**Gender (No. of Females (%))**	33 (94.29%)		27 (81.8%)	35 (89.74%)		0.258^◊^	
**BMI (No. > 30 Kg/m** ^**2**^ **(%))**	17 (48.57%)		19 (57.6%)	22 (56.41%)		0.714^◊^	
**No. of Subjects with Smoking History (%)**	3 (8.57%)		2 (6.1%)	3 (7.7%)		0.922^◊^	
**No. of Hypertensive Subjects (%)**	7 (20%)		5 (15.2%)	6 (15.38%)		0.829^◊^	
**No. of Diabetic Subjects (%)**	6 (17.14%)		4 (12.1%)	9 (23.1%)		0.477^◊^	
**Number of patients with other comorbidities (%)**							
**Pulmonary hypertension (%)**	3 (8.6%)		2 (6.1%)		3 (7.7%)	0.072^◊^	
**Cardiac problems (%)**	1 (2.86%)		1 (3.03%)		4 (10.26%)		
**Cancer (%)**	2 (5.7%)				1 (2.56%)		
**Rheumatoid arthritis (%)**	2 (5.7%)						
**Hepatosplenomegaly (%)**	2 (5.7%)						
**Others (%)**	2 (5.7%)				2 (5.13%)		

SD: Standard Deviation

BMI: body mass index

No.: Number

* Level of Significance at *p* < 0.05

^Θ^ One way ANOVA Test

^◊^ Chi-Square Test

### Clinical characteristics of recruited patients at baseline

#### Assessment of efficacy

- **Respiratory Function Assessment**: Respiratory function was assessed in terms of FEV_1_, FVC, the ratio between them, 6MWD and percentage of desaturation.
- **Inflammatory Markers**: KL-6, ESR and CRP were measured to indicate the level of inflammation and hence, the fibrosis prognosis in HP.
- At baseline, there was no statistically significant difference in those parameters among the three groups of the study as shown in Table 2.

#### Assessment of safety

- **Corticosteroids Side Effects**: To assess the corticosteroids’ side effects, blood glucose levels, blood pressure as well as liver function tests were measured at baseline to be able to compare to the after-treatment values. There was also no statistically significant difference in the safety parameters among the 3 study groups at baseline as shown in Table 2.

**Table 2:** Clinical and biochemical baseline data in the three tested groups (represented as (mean ± SD))

Parameter	Betamethasone (*N* = 35)	Dexamethasone (*N* = 33)	Prednisolone (*N* = 39)	***P***-Value^*^
**FEV**_**1**_**(**% predicted**)**	55.76 ± 20.02	53.53 ± 14.97	57.54 ± 20.51	0.714^Θ^
**FVC (**% predicted**)**	54.45 ± 16.6	55.27 ± 14.27	58.58 ± 21.53	0.578^Θ^
**FEV**_**1**_ **/FVC Ratio**	87.42 ± 10.69	85.19 ± 12.11	83.1 ± 12.98	0.306^Θ^
**6MWD (**m**)**	232.71 ± 77.1	237.35 ± 85.58	229.05 ± 95.41	0.922^Θ^
**Desaturation (**% SpO_2_**)**	9.46 ± 6.32	10.13 ± 8.95	9.73 ± 8.1	0.941^Θ^
**KL-6 (**U/ml**)**	723.22 ± 218.18	615.99 ± 146.66	669.44 ± 191.34	0.069^Θ^
**ESR (**mm**)**	58.1 ± 26.67	56.12 ± 27.97	59.96 ± 27.91	0.841^Θ^
**CRP (**mg/L**)**	18.26 ± 18.77	20.1 ± 18.79	19.48 ± 20.62	0.925^Θ^
**Blood Glucose (**mg/dl**)**	109.46 ± 35.55	104.12 ± 29.61	109 ± 48.13	0.823^Θ^
**Systolic Blood Pressure (**mmHg**)**	120.71 ± 9.63	120.91 ± 11.282	126.41 ± 12.028	0.052^Θ^
**Diastolic Blood Pressure (**mmHg**)**	79.57 ± 10.387	80 ± 9.186	83.85 ± 10.542	0.137^Θ^
**ALT (**U/L**)**	27.03 ± 17.64	27.82 ± 29.54	26.97 ± 14.87	0.983^Θ^
**AST (**U/L**)**	25.03 ± 9.56	25.36 ± 15.82	26.41 ± 11.3	0.882^Θ^

FEV_1_: forced expiratory volume after 1 s; 6MWD: six-minute walk distance

FVC: forced vital capacity; SpO_2_: peripheral capillary oxygen saturation

KL-6: krebs von den lungen − 6; CRP: C Reactive Protein

ESR: erythrocyte sedimentation rate; ALT: alanine aminotransferase

AST: aspartate aminotransferase

* Level of Significance at *p* < 0.05

^Θ^ One way ANOVA Test

### Therapeutic outcomes

#### Inflammatory markers

After 3 months of treatment, there was a significant effect of using different corticosteroids in KL-6 levels and ESR among the 3 study groups. Mean KL-6 levels were 554.48 U/ml ± 129.69 in the betamethasone group, 578.7 U/ml ± 91.92 in the dexamethasone group and 671.88 U/ml ± 162.63 in the prednisolone group (*p* = 0.001, One Way ANOVA test). Post-hoc analysis, showed that the significant difference was between prednisolone and betamethasone, prednisolone and dexamethasone groups (*p* = 0.001, *p* = 0.012 respectively, Bonferroni test). A multiple regression was conducted with age and gender as predictors with the KL-6 levels as the dependent variable. The results showed that age and gender did not explain the decrease in KL-6 levels across the treatment groups (β = 0.019, *p* = 0.847 and β = -0.073, *p* = 0.458 respectively) (supplementary table S2).

Reanalysis was done using analysis for covariance ANCOVA to adjust for baseline levels of KL-6 and a statistically significant difference was still seen between the treatment groups after 3 months of treatment (F = 16.586, *p* < 0.001). Furthermore, using the change in KL-6, it was found that the mean ± SD for all treatment groups is -65.81 ± 164.27. By comparing the means across the groups using ANOVA test there was a statistical significance between the 3 groups (*p* < 0.001). Post-hoc analyses showed a statistically significant difference between betamethasone/dexamethasone group and between betamethasone/prednisolone group (*p* = 0.001 and *p* < 0.001, respectively, Bonferroni test).

Mean ESR level in the betamethasone group was 38.77 mm ± 21.13, in the dexamethasone group was 30.06 mm ± 16.04 and in the prednisolone group was 42.3 mm ± 24.52 (*p* = 0.048, One Way ANOVA test). On post-hoc analysis, it was found that the significant difference is between dexamethasone group and prednisolone group (*p* = 0.047, Bonferroni test). A multiple regression was conducted with age and gender as predictors with the ESR levels as the dependent variable. The results showed that age and gender did not explain the decrease in ESR levels across the treatment groups (β = 0.124, *p* = 0.218 and β = -0.106, *p* = 0.294 respectively) (supplementary table S2).

**Fig. 1:**
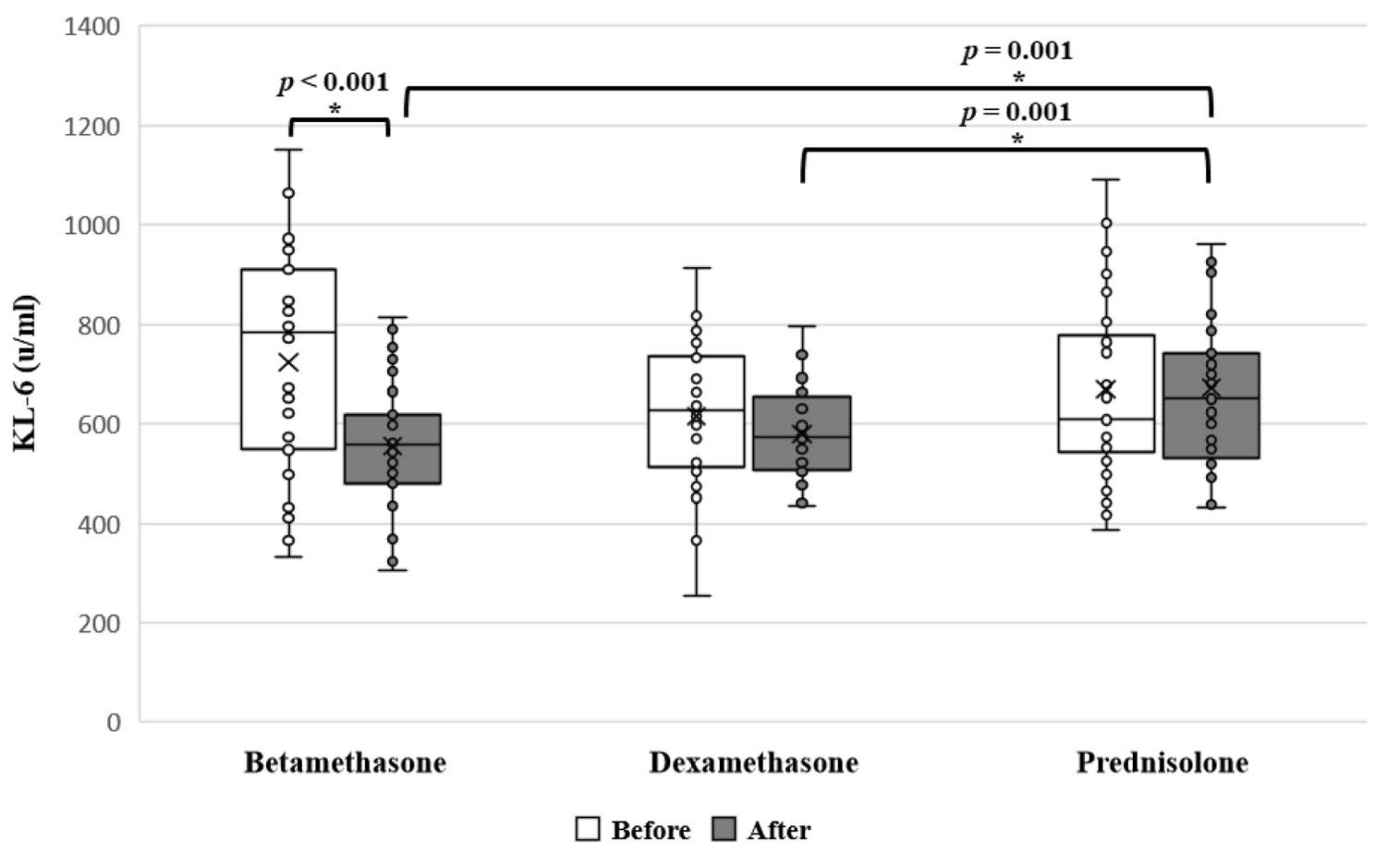
Effect of using different corticosteroids on KL-6 levels within the same group (Paired T-test, *P* < 0.05)

On testing the significant improvement within the same group, betamethasone group showed significant improvement in KL-6 levels post-treatment (*p* < 0.001, paired T-test) (Fig. 1).

**Fig. 2:**
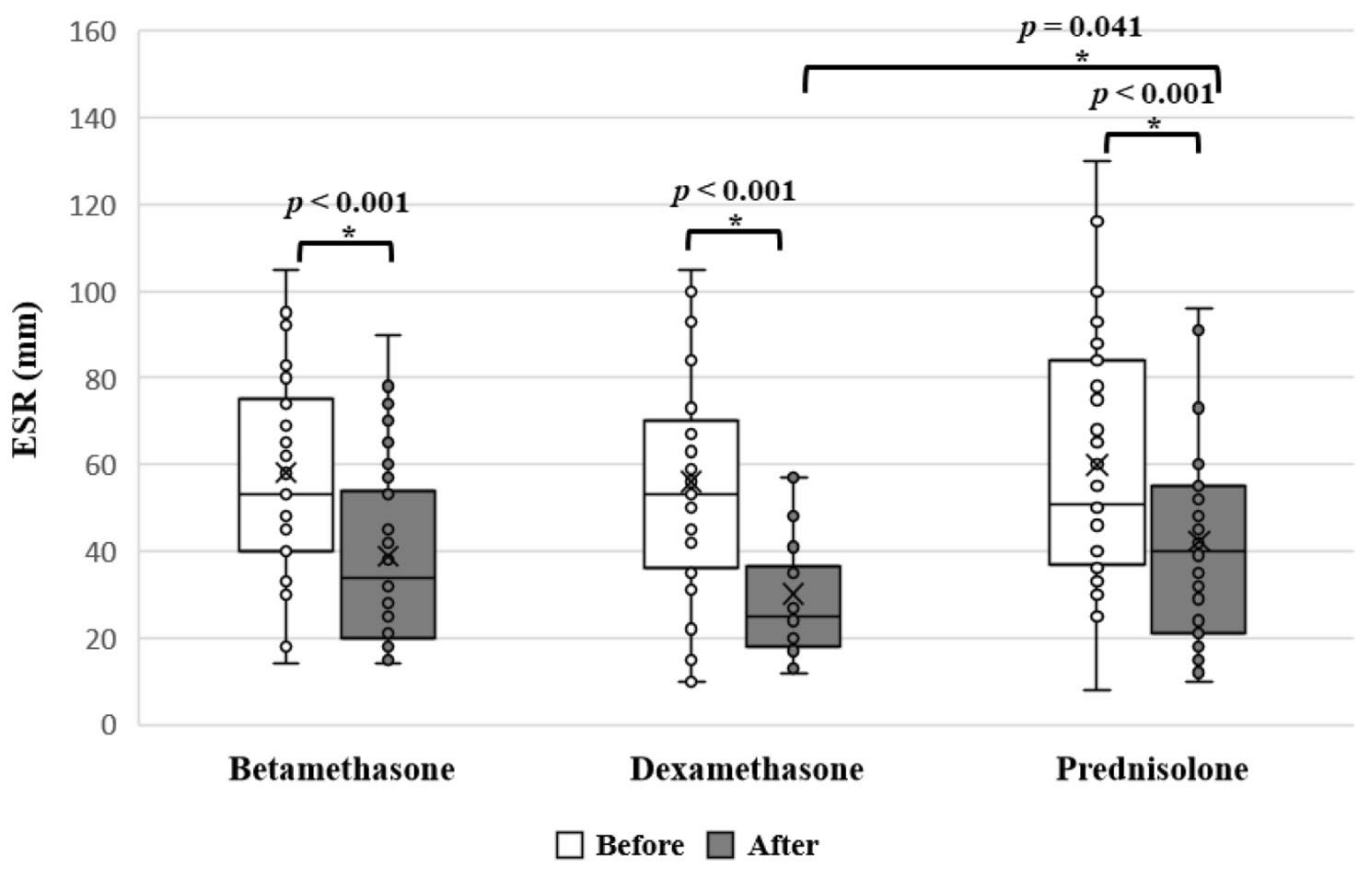
Effect of using different corticosteroids on ESR levels within the same group (Paired T-test, *P* < 0.05)

Also, there was a significant improvement within betamethasone, dexamethasone and prednisolone groups in ESR (*p* < 0.001, *p* < 0.001and *p* < 0.001, respectively, paired T-test) (Fig. 2) as well as CRP values (*p* = 0.003, *p* < 0.001 and *p* = 0.006, respectively, paired T-test).

**Table 3:** Effect of using 3 different corticosteroids on clinical and biochemical outcomes of the treatment groups (represented as (mean ± SD))

Parameter	Betamethasone (*N* = 35)	Dexamethasone (*N* = 33)	Prednisolone (*N* = 39)	***P***-Value^*^ (across groups)	***P***-Value^*^ (within the same group)
**FEV**_**1**_**(**% predicted**)**	58.34 ± 22.46	56.38 ± 16.1	59.59 ± 21.21	0.797^Θ^	**B**: 0.264^◊^ **D**: 0.082^◊^ **P**: 0.308^◊^
**FVC (**% predicted**)**	64.97 ± 18.54	63.59 ± 14.96	63.57 ± 19.46	0.932^Θ^	**B**: <0.001^◊^ **D**: <0.001^◊^ **P**: 0.016^◊^
**FEV**_**1**_ **/FVC Ratio**	85.37 ± 12.59	85.09 ± 10.42	82.41 ± 12.78	0.506^Θ^	**B**: 0.347^◊^ **D**: 0.965^◊^ **P**: 0.7^◊^
**6MWD (**m**)**	273.3 ± 69.9	268.94 ± 71.74	256.45 ± 103.15	0.672^Θ^	**B**: 0.001^◊^ **D**: 0.002^◊^ **P**: 0.002^◊^
**Desaturation (**% SpO_2_**)**	6.37 ± 5.61	5.48 ± 4.5	7.71 ± 6.64	0.256^Θ^	**B**: 0.001^◊^ **D**: 0.001^◊^ **P**: 0.05^◊^
**KL-6 (**U/ml**)**	554.48 ± 129.69	578.7 ± 91.92	671.88 ± 162.63	0.001^ΘΔ^	**B**: <0.001^◊^ **D**: 0.054^◊^ **P**: 0.921^◊^
**ESR (**mm**)**	38.77 ± 21.13	30.06 ± 16.04	42.3 ± 24.52	0.048^ΘΔ^	**B**: <0.001^◊^ **D**: <0.001^◊^ **P**: <0.001^◊^
**CRP (**mg/L**)**	12.29 ± 13.33	9.1 ± 4.79	15.34 ± 15.36	0.105^Θ^	**B**: 0.003^◊^ **D**: <0.001^◊^ **P**: 0.006^◊^

FEV_1_: forced expiratory volume after 1 s; 6MWD: six-minute walk distance

FVC: forced vital capacity; SpO_2_: peripheral capillary oxygen saturation

B: betamethasone group; D: dexamethasone group

P: prednisolone group; ^◊^ Paired T-test

* Level of Significance at *p* < 0.05; ^Θ^ One way ANOVA test

^Δ^ Adjustment for multiple comparisons: Bonferroni

#### Respiratory function assessment

**Fig. 3:**
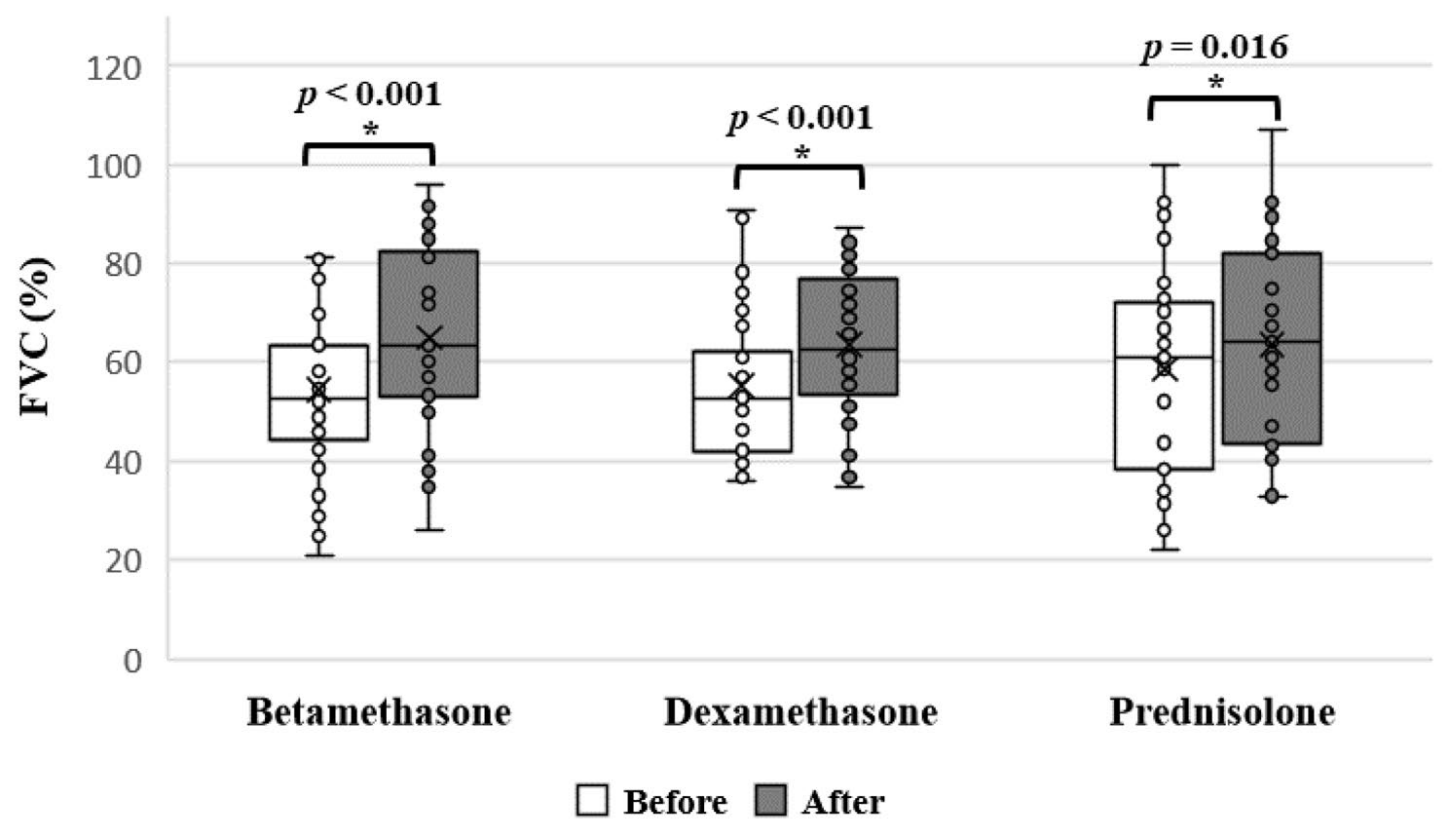
Effect of using different corticosteroids on FVC within the same group (Paired T-test, *P* < 0.05)

Regarding the values of FEV_1_, FVC, FEV_1_/FVC ratio, 6MWD and percentage of desaturation, no significant difference was found among the 3 different groups post-treatment (Table 3). However, on testing the improvement of the study subjects within the same group, a significant improvement was found in the FVC values in the betamethasone, dexamethasone and prednisolone groups (*p* < 0.001, *p* < 0.001 and *p* = 0.016, respectively, paired T-test) (Fig. 3). A multiple regression was conducted with age and gender as predictors with the change in FVC as the dependent variable. The results showed that age and gender did not explain the increase in FVC within the betamethasone group (β = -0.18, *p* = 0.378 and β = 0.168, *p* = 0.41 respectively), dexamethasone group (β = -0.19, *p* = 0.921 and β = 0.163, *p* = 0.397 respectively) and prednisolone group (β = 0.008, *p* = 0.964 and β = 0.117, *p* = 0.492 respectively) (supplementary table S2).

On further investigation, the change in FEV_1_, FVC and FEV_1_/FVC ratio was compared between the 3 groups post- treatment and there was no significant difference found (*p* = 0.774, *p* = 0.489 and *p* = 0.425, respectively, One Way ANOVA test).

**Fig. 4:**
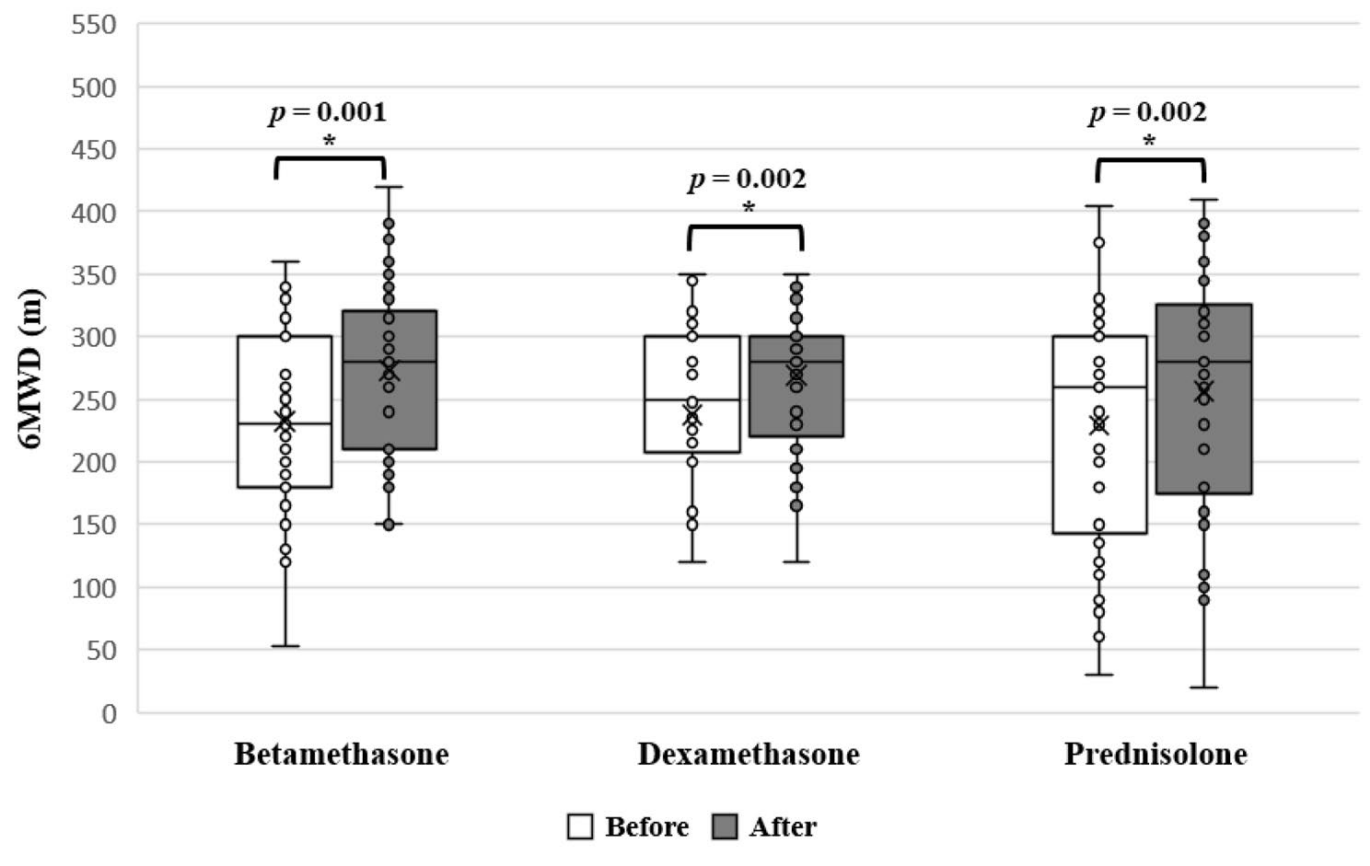
Effect of using different corticosteroids on 6MWD within the same group (Paired T-test, *P* < 0.05)

6MWD showed a significant improvement post-treatment within the betamethasone, dexamethasone and prednisolone groups (*p* = 0.001, *p* = 0.002 and *p* = 0.002, respectively, paired T-test) (Fig. 4). A multiple regression was conducted with age and gender as predictors with the change in 6MWD as the dependent variable. The results showed that age and gender did not explain the increase in 6MWD within the betamethasone group (β = 0.019, *p* = 0.925 and β = -0.201, *p* = 0.324 respectively), dexamethasone group (β = -0.098, *p* = 0.606 and β = -0.12, *p* = 0.529 respectively) and prednisolone group (β = -0.178, *p* = 0.0.295 and β = -0.194, *p* = 0.255 respectively) (supplementary table S2).

**Fig. 5:**
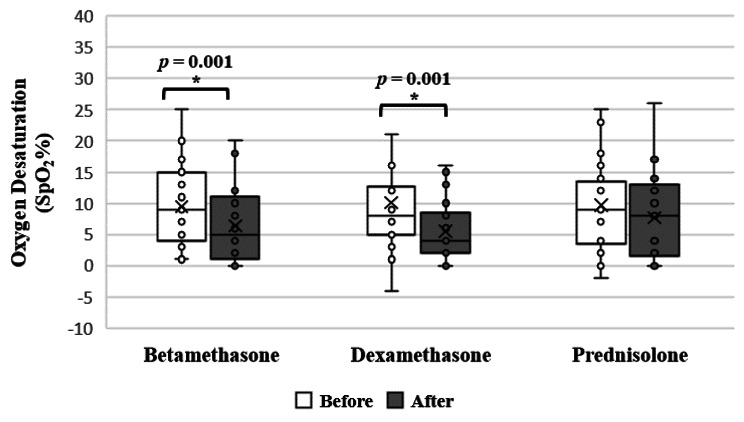
Effect of using different corticosteroids on Oxygen Desaturation percentage within the same group (Paired T-test, *P* < 0.05)

The percentage of desaturation has improved only within the betamethasone and dexamethasone groups (*p* = 0.001, *p* = 0.001, respectively, paired T-test) (Fig. 5). A multiple regression was conducted with age and gender as predictors with the change in SpO_2_ as the dependent variable. The results showed that age and gender did not explain the decrease in the change in SpO_2_ within the betamethasone group (β = 0.197, *p* = 0.284 and β = 0.338, *p* = 0.071 respectively) and dexamethasone group (β = -0.25, *p* = 0.18 and β = 0.349, *p* = 0.065 respectively) (supplementary table S2).

#### Assessment of safety

On measuring corticosteroids side effects among the 3 different groups, there was no statistically significant difference in the prevalence of side effects post-treatment (Table 4). On performing statistical testing within the same group, there was no significant difference found in the 3 groups of study except in the betamethasone group where the systolic blood pressure increased significantly post-treatment (*p* = 0.002, paired T-test) where 8 patients of the betamethasone group had increased systolic blood pressure (range 140–160 mmHg) and 27 patients had systolic blood pressure of 130 mmHg and lower.

**Table 4:** Parameters measuring corticosteroids side effects post-treatment (represented as (mean ± SD))

Parameter	Betamethasone (*N* = 35)	Dexamethasone (*N* = 33)	Prednisolone (*N* = 39)	***P***-Value^*^	
**Blood Glucose (**mg/dl**)**	114.54 ± 34.83	109.61 ± 34.49	111.9 ± 35.24	0.843^Θ^	
**Systolic Blood Pressure (**mmHg**)**	127.74 ± 12.94	122.73 ± 11.798	125.77 ± 12.489	0.251^Θ^	
**Diastolic Blood Pressure (**mmHg**)**	83.83 ± 12.118	80 ± 10	81.92 ± 10.363	0.352^Θ^	
**ALT (**U/L**)**	32.73 ± 30.44	28 ± 26.51	26.31 ± 18.29	0.538^Θ^	
**AST (**U/L**)**	31.02 ± 26.93	22.42 ± 11.99	27.69 ± 12.94	0.161^Θ^	

ALT: alanine aminotransferase; AST: aspartate aminotransferase

* Level of Significance at *p* < 0.05; ^Θ^ One way ANOVA Test

### Cost-effectiveness analysis

All costs are calculated based on 2021 prices in Egyptian pound (EGP). As oral prednisolone is the conventional standard treatment of HP in Egypt [37]; it was used to compare the costs with the other 2 selected corticosteroids to make a cost effectiveness decision. The costs expended by the patients include the cost of medications (for HP or for adverse drug reactions by corticosteroids), medication administration costs (for parenteral corticosteroids), costs of any laboratory tests done, costs of transportation of the patient, cost of hospital stay due to HP or complications to treatment and the cost of days-off from work for the patient (supplementary table S1).Upon calculating the total costs paid by the patients to complete a treatment period of 3 months using the selected corticosteroids, it was found that the total cost of using betamethasone weekly injections is EGP 70,096 to get 23.33% decrease in KL-6 levels, the total cost of using dexamethasone weekly injections is EGP 69,756 to get 6.06% decrease in KL-6 levels while the cost of prednisolone daily tablets is EGP 74,375 with no decrease in KL-6 levels. When comparing the proposed new interventions (betamethasone and dexamethasone) with the conventional intervention (prednisolone) by calculating the ICERs (-180.59 EGP/1% decrease in KL-6, -719.48 EGP/1% decrease in KL-6, respectively) (Table 5) and placing them in the cost-effectiveness plane [34], ICERs fell in the less costly, more effective quadrant which is a dominant decision to take. Both betamethasone and dexamethasone were found to be cost-effective compared to prednisolone. On further investigation of cost-effectiveness, the costs were compared to the increase in FVC_6_, there was also a dominant decision in favor of betamethasone and dexamethasone due to decreased cost against the increased effect (Table 5). However, on calculating ICERs based on 6MWD, ICER of dexamethasone compared to prednisolone fell in the less costly, less effective quadrant (Table 5).

However, to determine whether betamethasone or dexamethasone is the most cost-effective ICER was calculated between betamethasone and dexamethasone (19.65 EGP/1% decrease in KL-6), (76.96 EGP/1% increase in FVC_6_) and (77.46 EGP/1% increase in 6MWD) (Table 5).This leaves us in more costly, more effective quadrant of the cost-effectiveness plane [34] and a trade-off decision needs to be made. Since the difference in cost between betamethasone and dexamethasone is small compared to the total cost (EGP 340) and the improvement with betamethasone is higher, which is a good value for the spent cost [34] implying that betamethasone is the best intervention in terms of cost against the effect.

**Table 5:** Incremental cost-effectiveness ratios (ICER) of 3 different corticosteroids

	Betamethasone	Dexamethasone	Prednisolone	ICER Betamethasone	ICER Dexamethasone	ICER Beta./Dexa.
**Cost**	70095.5	69,756	74,375			
**KL-6 (%decrease)**	23.33%	6.06%	-0.365%	-180.6	-719.48	19.65
**FVC (%increase)**	19.31%	15.04%	8.53%	-397.21	-709.14	79.69
**6MWD (%increase)**	17.69%	13.31%	14.99%	-1580.45	2757.08	77.46

## Discussion

Hypersensitivity pneumonitis (HP) is the third most frequent ILD [38], it is stimulated by inhalation of various antigens from organic dust leading to immunologically mediated inflammatory reaction and hence fibrosis [39]. In chronic HP, fibrotic changes such as reticular opacities, traction bronchiectasis, and honeycombing may be observed. While these changes are often irreversible, effective treatment can help in stabilizing these changes or slowing their progression. The interpretation of HRCT changes should be done in conjunction with clinical assessment, including symptoms, lung function tests, antigen exposure history and in our study, we added the inflammatory markers specially KL-6 as a more objective way to detect the decrease in inflammatory response and accordingly the fibrosis. KL-6 is a very useful biomarker in the diagnosis and prognosis of different types of ILDs [39]. In, literature, KL-6 was found to be more reliable in measuring ILDs prognosis than in diagnosis [40]. 70–100% of patients with ILD had abnormal KL-6 levels, as opposed to only 10% of patients with pneumonia, asthma, or chronic obstructive pulmonary disorder and 28% of patients with active pulmonary tuberculosis [15]. A study performed in 2006 [41], showed that KL-6 is the most reliable diagnostic and prognostic parameter with accuracy of 77.8% compared to 66.7% of FVC and 59.3% of partial oxygen pressure (PaO_2_), moreover, it was found to be 90% sensitive compared to 70.6% sensitivity provided by FVC and 58.8% provided by PaO_2_. A study conducted in 2012 [42], correlated the levels of serum KL-6 to the percentage of lymphocytes in bronchoalveolar lavage fluid showing that lymphocyte infiltration was more persistent in HP and hence, KL-6 levels are remarkably higher in HP and very useful in HP diagnosis. Moreover, a study conducted in 2014, proved that HP was the most sensitive ILD to seasonal changes in KL-6 levels [43]. A study conducted in 2015 [44], concluded that there is an increased risk of missing some types of ILDs when relying only on PFTs especially that clinical symptoms are non-specific and of late onset; it was found that 53% of the study subjects had normal FVC values despite remarkable ILD on HRCT. A study done by *Okamoto et al.* on patients with hypersensitivity pneumonitis showed a significant decrease in KL-6 levels after only 1 month of steroid therapy [39]. A systematic review and metanalysis done by *Tao Zahng et al.* concluded that the higher levels of KL-6 in ILD patients, the more severe and more progressive the ILD will be. Also, the high levels of KL-6 indicated a higher mortality rate and poor outcomes in ILD patients [45]. These findings were aligned to the present study by using KL-6 as the primary outcome to measure disease prognosis. Despite the usefulness of KL-6 in the diagnosis and treatment monitoring of chronic HP, its use has not been widely accepted due to the variability in its levels across different populations which may be related to differences in living environments as well as the differences in immune responses in different ethnic groups [46].

The main therapeutic intervention for treatment of HP is reducing inflammation by corticosteroids and preventing antigen exposure [47, 48]. Yet, a cohort study conducted in 2019 [38], stated that the use of corticosteroids showed no effect on the management of fibrotic HP nor the pulmonary function tests. These findings were aligned with results from Morisset et al. [49], Adegunsoye et al. [50] and Gimenez et al. [51]. However, these results are not consistent with the present study; where treatment with 3 different corticosteroids, namely betamethasone, dexamethasone and prednisolone, showed significant improvement in FVC, 6MWD after 3 months of treatment. Moreover, betamethasone and dexamethasone improved the oxygen desaturation occurring after exertion. These results are supported by a study conducted in Egypt and published in 2021 [52], which showed improvement in FEV_1_, FVC, 6MWD and oxygen saturation upon treatment with prednisolone.

To the best of our knowledge this is the first study comparing the use of pulse versus sustained steroid treatment and comparing the use of prednisolone versus betamethasone or dexamethasone in management of HP. A systematic review conducted in 2018 comparing the use of single dose parenteral corticosteroids to the regular oral corticosteroid regimen in decreasing asthma relapse [53], found that there was no significant difference between the two regimens. However, Lahn et al. [54], stated that the single use of parenteral corticosteroid is a more viable alternative in preventing asthmatic exacerbations than the regular oral course of corticosteroids. Moreover, Edalatifard et al. [55], stated that using pulse corticosteroids had a significant role in improving pulmonary involvement, oxygen saturation, dyspnea, heart rate, respiratory rate, temperature and inflammatory markers in treatment of hospitalized COVID-19 patients. The latter findings are consistent with our study as the use of pulse corticosteroids was superior to the conventional oral dosing in decreasing inflammatory markers (KL-6 and ESR). Sinha et al. [56], favored the use of pulse corticosteroids in terms of lower cumulative toxicity compared to sustained steroid therapy even at a lower quantitative dose.

Chan et al. [57], compared single dose IM betamethasone to oral prednisolone in preventing acute asthmatic relapses and found that betamethasone use is safe and leads to an improvement in the early outcomes. These comes aligned with the results of the present study where betamethasone has a more favorable effect on the improvement of KL-6 levels in treatment of HP. Meyer et al. [58], found that dexamethasone is an effective choice to be used over prednisolone in the treatment of mild to moderate asthmatic exacerbations. This finding is consistent with our study where dexamethasone is found to be better than prednisolone in improving inflammatory markers as KL-6 and ESR in the management of HP.

To maximize patient care; the use of medications should be rational, evidence-based, safe, clinically appropriate and cost-effective [59]. Cost-effectiveness analysis (CEA) is a type of economic evaluation that uses natural health units as measurement of outcomes such as life years gained, avoided deterioration or symptom-free days [60]. For the comparator interventions total costs and total benefits are measured to calculate the average cost-effectiveness ratio; mean value of costs divided by mean value of effect, to consider which intervention is worth to pay for [24]. In this study, where a difference in the effectiveness was proven; it gave us the chance to perform CEA in terms of costs versus benefit to determine which treatment is the most cost effective. The results of this study showed that betamethasone is the most cost-effective medication although it was slightly of higher price compared to dexamethasone. The slight increase in the price was for by the greater difference in effect However, a meta-analysis conducted in 2021 [61], found that both dexamethasone and betamethasone have comparable efficacy in treating neonatal-relevant outcomes for women with risk of preterm birth unlike our study, furthermore dexamethasone is much more cheaper making it the cost-effective choice when used in preterm birth. Another study performed in 2012 [62], showed that dexamethasone was more cost-effective than prednisone in the treatment of pediatric asthma exacerbations with total savings of $3500 and decreased number of emergency department visits and hospital admissions. This finding is consistent with the results of our study that dexamethasone is a more cost-effective choice than prednisolone in terms of costs and benefits.

Limitations of this study include being short termed, single center and the lack of quality-of-life assessment. In addition, intramuscular and intravenous injections, despite being more effective, are not favored by patients. Another limitation was the lack of modelling variability in the cost-effectiveness analysis which may have an impact on the interpretation. Therefore. longer term trials on HP and other types of ILDs response to different corticosteroids might be useful along with investigating the reason behind the difference in the patients’ response to variable corticosteroids.

## Conclusion

In summary, this study has shed light on the importance of the role of inflammatory biomarkers as KL-6 in diagnosis of hypersensitivity pneumonitis and consequently the monitoring of therapeutic effect of the used medications for the treatment of HP. Both betamethasone and dexamethasone have shown to be more effective than prednisolone in terms of reduction in KL-6 and hence improvement in the inflammatory reaction and clinical features of HP patients. However, the three investigated corticosteroids had a comparable effect on the change in pulmonary function tests as FEV_1_, FVC, 6MWD and percentage of oxygen desaturation. The three corticosteroids had a comparable satisfactory safety profile with no significant elevation in blood glucose levels, blood pressure or liver enzymes. Moreover, CEA was conducted where the direct costs were compared to the change in KL-6 levels in the three treatment groups. The resulting ICERs have proved that both betamethasone and dexamethasone are more cost-effective than prednisolone. The latter result offers more benefit to HP patients as they will pay less money for better improvement in their conditions.

### Electronic supplementary material

Below is the link to the electronic supplementary material.

Supplementary Material 1

## Data Availability

The datasets used and/or analysed during the current study are available from the corresponding author on reasonable request.
