# Supplementary material for: The effectiveness and pharmacoeconomic study of using different corticosteroids in the treatment of hypersensitivity pneumonitis
Source: BMC Pulm Med. 2024 Feb 15;24:87. doi: 10.1186/s12890-024-02896-z (PMC10870560; doi:10.1186/s12890-024-02896-z)
Supplement: Supplementary file 1 — Supplementary Material 1 [file 12890_2024_2896_MOESM1_ESM.docx]

**Supplementary Material**

| **Table S1.** Detailed costs of interventions across the three treatment groups. | | | | | |
| --- | --- | --- | --- | --- | --- |
|  | **Medication & Administration** | **Laboratory Tests** | **Transportation** | **Hospital stays** | **Days-off** |
| **Betamethasone** | 39207.5 | 19220 | 7268 | - | 4400 |
| **Dexamethasone** | 34525.5 | 16330.5 | 9600 | - | 9300 |
| **Prednisolone** | 37413 | 21240 | 9922 | - | 5800 |
| All costs are represented as the collective cost of the treatment group in EGP | | | | | |

| **Table S2.** Univariate and Multivariate Multiple Regression Analyses on the Different Clinical Outcomes. | | | | | | | |
| --- | --- | --- | --- | --- | --- | --- | --- |
| **Dependant Variable** | **Independent Variable** | **Univariate** | | | **Multivariate** | | |
|  |  | **Standardized Coefficient Beta** | **95% Confidence Interval** | **P-Value** | **Standardized Coefficient Beta** | **95% Confidence Interval** | **P-Value** |
| **KL-6 (U/ml)** | **Betamethasone group** | - 0.248 | -131.11 – -18.26 | 0.01 | -0.385 | -177.92 – -53.61 | <0.001 |
|  | **Dexamethasone group** | - 0.123 | -96.37 – 21.06 | 0.206 | -0.312 | -158.75 – -32.11 | 0.004 |
|  | **Age** | 0.011 | -2.29 – 2.58 | 0.907 | 0.019 | -2.17 – 2.64 | 0.847 |
|  | **Gender** | - 0.068 | -116.74 – 56.06 | 0.488 | -0.073 | -118.96 – 53.99 | 0.458 |
| **ESR (mm)** | **Betamethasone group** | 0.046 | -6.74 – 10.9 | 0.641 | -0.067 | -12.81 – 6.72 | 0.538 |
|  | **Dexamethasone group** | -0.228 | -19.3 – -1.84 | 0.018 | -0.269 | -22.43 – -2.53 | 0.014 |
|  | **Age** | 0.104 | -0.168 – 0.565 | 0.285 | 0.124 | -0.142 – 0.613 | 0.218 |
|  | **Gender** | -0.038 | -15.7 – 10.53 | 0.697 | -0.106 | -20.81 – 6.37 | 0.294 |
| **FVC (% predicted)** | **Treatment group** | **Betamethasone Group** | | | | | |
|  | **Age** | -0.095 | -0.49 – 0.29 | 0.586 | -0.18 | -0.65 – 0.255 | 0.378 |
|  | **Gender** | 0.077 | -15.71 – 24.54 | 0.658 | 0.168 | -13.81 – 32.98 | 0.41 |
|  | **Treatment group** | **Dexamethasone Group** | | | | | |
|  | **Age** | 0.031 | -0.41 – 0.481 | 0.863 | -0.019 | -0.492 – 0.446 | 0.921 |
|  | **Gender** | 0.157 | -6.28 – 15.93 | 0.383 | 0.163 | -6.88 – 16.88 | 0.397 |
| **6MWD (m)** | **Treatment group** | **Betamethasone Group** | | | | | |
|  | **Age** | -0.082 | -2.29 – 1.42 | 0.638 | 0.019 | -2.05 – 2.25 | 0.925 |
|  | **Gender** | -0.192 | -145.97 – 42.21 | 0.27 | -0.201 | -165.2 – 56.27 | 0.324 |
|  | **Treatment group** | **Dexamethasone Group** | | | | | |
|  | **Age** | -0.135 | -2.67 – 1.22 | 0.453 | -0.098 | -2.59 – 1.54 | 0.606 |
|  | **Gender** | -0.151 | -69.69 – 28.77 | 0.403 | -0.12 | -68.78 – 36.08 | 0.529 |
| **Desaturation**  **(%SpO_2_)** | **Treatment group** | **Betamethasone Group** | | | | | |
|  | **Age** | 0.367 | 0.016 – 0.293 | 0.068 | 0.197 | -0.072 – 0.238 | 0.284 |
|  | **Gender** | 0.437 | 2.562 – 16.35 | 0.09 | 0.338 | -0.66 – 15.27 | 0.071 |
|  | **Treatment group** | **Dexamethasone Group** | | | | | |
|  | **Age** | -0.142 | -0.347 – 0.154 | 0.437 | -0.25 | -0.423 – 0.083 | 0.18 |
|  | **Gender** | 0.272 | -1.507 – 10.82 | 0.133 | 0.349 | -0.404 – 12.46 | 0.065 |
